# Cost-effectiveness of dapagliflozin for the treatment of heart failure: a systematic review

**DOI:** 10.3389/fphar.2025.1572289

**Published:** 2025-05-23

**Authors:** Zehui Jiang, Dong xiao Chen, Cai Xiao, Ying Fu, Jun Zhang

**Affiliations:** ^1^ Faculty of Economics and Management, Jiangxi University of Chinese Medicine, Nanchang, China; ^2^ Manchester Centre for Health Economics, Division of Population Health, Health Service Research and Primary Care, University of Manchester, Manchester, United Kingdom; ^3^ Breast Center, The First Affiliated Hospital of Nanchang University, Nanchang, China; ^4^ Department of Cardiovascular Medicine, The 908^th^ Hospital of Chinese People’s Liberation Army Joint Logistic Support Force, Nanchang, China

**Keywords:** dapagliflozin, heart failure, economic evaluation, cost-effectiveness, systematic review

## Abstract

**Objective:**

This study aims to synthesize evidence on the cost-effectiveness of dapagliflozin for heart failure (HF) with all ejection fractions (EF), Including heart failure with reduced ejection fraction (HFrEF), heart failure with mildly reduced ejection fraction (HFmrEF) and heart failure with preserved ejection fraction (HFpEF).

**Methods:**

Literature searches were conducted in English-language databases (PubMed, web of science, Embase, Cochrane Library) and Chinese-language databases (CNKI, Wanfang Data, and Chongqing VIP) to identify studies of dapagliflozin for heart failure. The search was current to 3 October 2024.

**Results:**

Twenty-eight studies were identified in the systematic review and the overall quality was accepted. Studies were conducted across 15 countries including China, UK, US, Japan, South Korea, Singapore, Thailand, Australia, Egypt, Colombia, Philippines, Qatar, Canadian, German, and Spanish. Cost-effectiveness analyses of dapagliflozin were performed for HFrEF patients in all countries, HFpEF patients in the US and China, HFpEF/HFmrEF patients in the UK, Germany, Spain and China and HF patients in the UK, US, Korea and Thailand. Except for one study in Thailand, all studies showed that dapagliflozin is cost-effective. One study in Korea showed that the cost-effectiveness of dapagliflozin in patients with left ventricular EF (LVEF)≤40% was more pronounced than LVEF >40%. Four studies (two HFrEF and one HFpEF in the US and one HFrEF in China) showed that dapagliflozin was more cost-effective than empagliflozin. In the nine diabetes subgroup analyses, seven results showed that dapagliflozin was more cost-effective for patients with diabetes. The incremental cost-effectiveness ratios (ICER)were most sensitive to the cost of dapagliflozin and cardiovascular mortality in the uncertainty analysis.

**Conclusion:**

Dapagliflozin is cost-effective in the treatment of HF with all ejection fractions. The cost-effectiveness of patients with LVEF≤40% (HErEF)was more pronounced than LVEF >40% (HFpEF and HFmrEF). Compared to empagliflozin, dapagliflozin may be more cost-effective.

## Introduction

Heart failure is a complex clinical syndrome resulting from abnormal changes in the structure and/or function of the heart caused by various factors, leading to impaired ventricular systolic and/or diastolic function. Heart failure imposes a substantial economic burden on healthcare systems and patients, encompassing both direct medical costs (e.g., medications, hospitalizations) and indirect costs (e.g., productivity loss). The global economic burden attributable to HF has been estimated to be US$108 billion per annum, of which direct costs (e.g., medications and healthcare services) and indirect costs (e.g., loss of productivity caused by morbidity and mortality) accounted for around 60% and 40%, respectively (Cook et al., 2014). Therefore, it is imperative to perform an economic evaluation of HF therapies in order to alleviate its social and economic burden.

Heart failure can be categorized into three basic types based on left ventricular ejection fraction (LVEF): “heart failure with reduced ejection fraction (HFrEF, LVEF≤40%)”, “ heart failure with mildly reduced ejection fraction (HFmrEF, LVEF 41%–49%)”, and “heart failure with preserved ejection fraction (HFpEF, LVEF≥50%)” (Bozkurt et al., 2021; Lam and Solomon, 2021). Studies have shown that sodium-glucose transporter 2 inhibitors (SGLT2 inhibitors) can significantly reduce the composite endpoint risk of cardiovascular death or heart failure hospitalization in HF patients, regardless of LVEF levels (Vaduganathan et al., 2022). In the latest 2023 ESC guidelines for heart failure (Mcdonagh et al., 2024), regarding the recommendations for pharmacological treatment of chronic heart failure, sodium-glucose transporter 2 inhibitors (SGLT2 inhibitors) have emerged as the sole currently recommended medication for the treatment of heart failure with all ejection fractions. As one of the representative drugs of SGLT2 inhibitors, dapagliflozin can exert cardioprotective effects by inhibiting sympathetic nervous system excitation, improving myocardial energy metabolism, promoting urinary sodium excretion, and inhibiting inflammatory reactions (Sarafidis et al., 2021). In addition, dapagliflozin can lower blood pressure, reduce volume load, lower cardiac load, and alleviate HF symptoms in patients (Packer et al., 2020). The DAPA-HF trial found that dapagliflozin can reduce the incidence of cardiovascular death and hospitalization in HFrEF patients (Colombo et al., 2020; McDonagh et al., 2021). The results of the DELIVER study (Solomon et al., 2022) showed that the use of dapagliflozin can significantly reduce the risk of hospitalization for heart failure in HFpEF patients. It can be seen that dapagliflozin has significant therapeutic effects on patients with heart failure, and it is necessary to study its cost-effectiveness. Previous studies conducted systematic reviews on the cost-effectiveness of dapagliflozin in patients with HFrEF (Wu et al., 2022; Mohammadnezhad et al., 2022; Rezapour et al., 2023). However, there has been no systematic review on the cost-effectiveness of dapagliflozin in patients with HFmrEF and HFpEF. Therefore, this study is the first systematic review to summarize the research results on the cost-effectiveness of dapagliflozin in the treatment of heart failure with all ejection fraction, providing valuable reference for clinical and health decision-making.

## Methods

### Search strategy

Following the Preferred Reporting Items for Systematic Reviews and Meta-Analyses (PRISMA), we used English databases (PubMed, Web of science, Embase, Cochrane Library) and Chinese databases (CNKI, WanFang Data, and ChongQing VIP) to search articles on cost-effectiveness of dapagliflozin for HF. The literature search was ended on 3 October 2024, and the detail of the search is presented in Supplementary Table S1.

### Eligibility criteria

Our eligibility criteria were: 1) Population was patients with HF; 2) Intervention was dapagliflozin, and comparison was not limited; 3) Economic evaluation including cost-effectiveness analyses or cost–benefit analyses or cost-utility analyses or cost-minimization analyses.

### Study selection

Two independent investigators screened all retrieved studies for eligibility, with discrepancies resolved through discussion. All researchers resolved any conflicts through discussion. Full texts were selected according to eligibility criteria. Conference proceedings and abstracts were excluded.

### Quality assessment

The Consolidated Health Economic Evaluation Reporting Standards (CHEERS) checklist was applied for the quality assessment of studies. The CHEERS checklist contains 28 items. Each item was scored as having met the criteria (“1”), not at all (“0”), or not applicable (NA). Studies were considered as high quality if scores more than 75% of checklist items.

### Data extraction and analysis

Data were extracted independently by two reviewers (DXC and CX). Conflict resolution was resolved through discussion with a third reviewer and 2 reviewers. The data extracted from each study includes basic information (author, country, population, intervention, comparison), method and results. We analyzed and compared the basic characteristics, economic evaluation outcomes, uncertainty analysis and subgroup analysis of the studies.

For better comparison and more accurate judgment, all costs and ICERs were converted to US dollars by using the “CCEMG-EPPI-Centre Cost Converter” (Version 1.7).

## Results

### Database search findings

**FIGURE 1:**
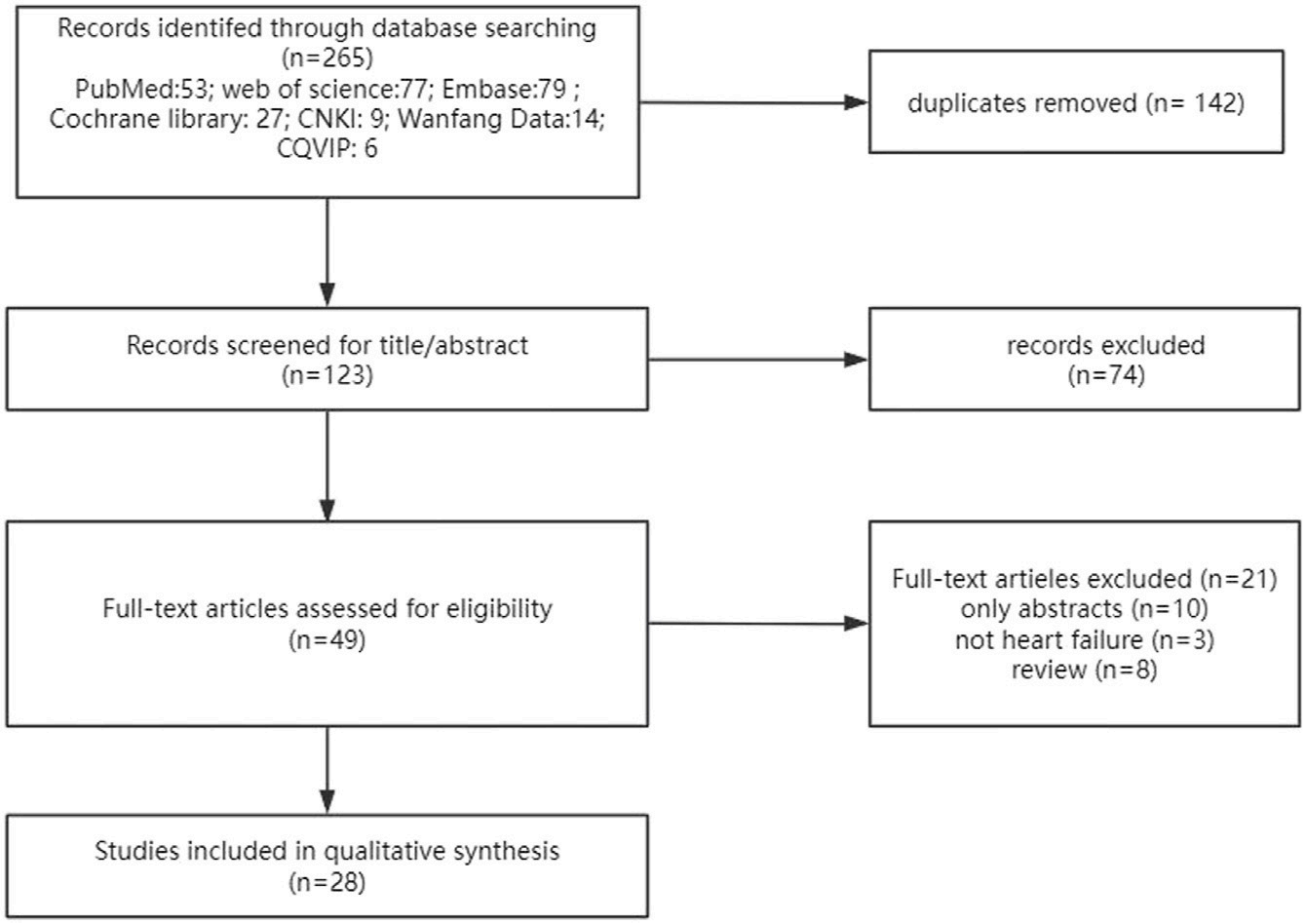
Flowchart of literature search. CNKI: China National Knowledge Infrastructure database; CQVIP: Chongqing VIP database.

As shown in Figure 1, 265 studies were retrieved in the primary search, and after removing duplicates, 123 studies remained. Then, 74 studies were removed based on the title and abstract, and 49 studies remained to be reviewed by full text. Finally, 28 economic evaluations remained.

### Basic characteristics

Table 1 summarizes characteristics of each included study. Among the 28 included studies, 10 were conducted in China (Lu H. et al., 2023; Tang and Sang, 2023; Lin et al., 2023; Jiang et al., 2021; Yao et al., 2020; Liao et al., 2021; Chen et al., 2022; Xu et al., 2023; Xia et al., 2022; Lu D. et al., 2023), 6 in the United States (Nechi et al., 2023; Parizo et al., 2021; Isaza et al., 2021; Nguyen et al., 2023; Bhatt et al., 2024; Rane et al., 2023), 4 in the UK (Booth et al., 2023; Miller et al., 2023; Davis et al., 2024; McEwan et al., 2020), 3 in Germany (Booth et al., 2023; Miller et al., 2023; McEwan et al., 2020), 3 in Spain (Booth et al., 2023; Miller et al., 2023; McEwan et al., 2020), 2 in South Korea (Kim et al., 2024; Liao et al., 2021), 2 in Thailand (Kongmalai et al., 2025; Krittayaphong and Permsuwan, 2021), 2 in Australia (Savira et al., 2021; Liao et al., 2021), 1 in Singapore (Liao et al., 2021), 1 in Japan (Liao et al., 2021), 1 in Qatar (Abushanab et al., 2024), 1 in Canada (Miller et al., 2023), 1 in Egypt (Abdelhamid et al., 2022),1 in Colombia (Gil-Rojas et al., 2022) and 1 in Philippines (Mendoza et al., 2021). Most studies applied a healthcare system perspective (n = 19) (Lu H. et al., 2023; Kim et al., 2024; Abdelhamid et al., 2022; Gil-Rojas et al., 2022; Krittayaphong and Permsuwan, 2021; Tang and Sang, 2023; Miller et al., 2023; Nechi et al., 2023; Savira et al., 2021; Isaza et al., 2021; Nguyen et al., 2023; Abushanab et al., 2024; Jiang et al., 2021; Liao et al., 2021; Bhatt et al., 2024; Rane et al., 2023; Chen et al., 2022; Xu et al., 2023; Lu D. et al., 2023), six studies used a payer perspective (n = 6) (Booth et al., 2023; Parizo et al., 2021; Davis et al., 2024; McEwan et al., 2020; Yao et al., 2020; Xia et al., 2022), one study applied a healthcare provider perspective (Mendoza et al., 2021), one study reported a societal perspective (Kongmalai et al., 2025), and one study used a national insurance perspective (Lin et al., 2023).

**TABLE 1:** General characteristics of the included studies.

Author	Country	Perspective	Model	Target population	Age	Intervention	Comparison	Cost	Length of cycle	Time horizon	Discount rate (%)	Health outcomes	Sensitivity analysis
Booth et al. (2023)	United Kingdom	payers	Markov	HFpEF HFmrEF	一	DAPA + SOC	SOC	direct cost	1 month	lifetime	3.5	QALY	1way,PSA
	Germany										3		
	Spain										3		
Lu et al. (2023a)	China	healthcare system	Markov	HFpEF	72	DAPA + SOC EMPA + SOC	SOC	direct cost	3 months	20 years	5	QALY	1way,PSA
Kim et al. (2024)	South Korea	healthcare system	Markov	HF	66	DAPA + SOC	SOC	direct cost	1 month	lifetime	4.5	QALY	1way,PSA
Abdelhamid et al. (2022)	Egypt	healthcare system	Markov	HFrEF	≥18	DAPA + SOC	SOC	direct cost	1 month	lifetime	一	QALY	1way,PSA
Gil-Rojas et al. (2022)	Colombia	health system	Markov	HFrEF	≥18	DAPA + SOC	SOC	direct cost	1 month	5 years	5	QALY	1way,PSA
Kongmalai et al. (2025)	Thailand	society	Markov	T2D-HF	60	DAPA + SOC CANA + SOC EMPA + SOC	SOC	direct cost	3 months	lifetime	3	QALY	1way,PSA
Krittayaphong and Permsuwan, (2021)	Thailand	healthcare system	Markov	HFrEF	65	DAPA + SOC	SOC	direct cost	3 months	lifetime	3	QALY	1way,PSA
Mendoza et al. (2021)	Philippines	public healthcare provider	Markov	HFrEF	55	DAPA + SOC	SOC	direct cost	1 year	lifetime	3	QALY	1way,PSA
Tang and Sang, (2023)	China	public healthcare system	Markov	HFpEF HFmrEF	65	DAPA + SOC	SOC	direct cost	3 months	15 years	5	QALY	1way,PSA
Miller et al. (2023)	United Kingdom	healthcare systems	Markov	HFrEF	一	DAPA + SOC	SOC	direct cost	1 month	lifetime	3.5	QALY	1way,PSA
	Canada										1.5		
	Germany										3		
	Spain										3		
Nechi et al. (2023)	United States	healthcare system	Markov	HFrEF	65	DAPA + SOC	EMPA + SOC	direct cost	1 year	lifetime	3	QALY	1way,PSA
Lin et al. (2023)	China	national insurance	Markov	HFpEF HFmrEF	67	DAPA + SOC	SOC	direct cost	1 month	lifetime	5	QALY,LY	1way,PSA
Savira et al. (2021)	Australia	public healthcare system	Markov	HFrEF	66	DAPA + SOC	SOC	direct cost	1 year	lifetime	5	QALY, YoLS	1way,PSA
Parizo et al. (2021)	United States	healthcare payor	Markov	HFrEF	一	DAPA + SOC	SOC	direct cost	1 month	lifetime	3	QALY	1way,2way,PSA
Isaza et al. (2021)	United States	healthcare sector	Markov	HFrEF	66	DAPA + SOC	SOC	direct cost	1 month	lifetime	3	QALY	1way,PSA
Davis et al. (2024)	United Kingdom	payer	Markov	HF	69.37	DAPA + SOC	SOC	direct cost	1 month	lifetime	3.5	QALY	1way,2way,PSA
McEwan et al. (2020)	United Kingdom	payers	Markov	HFrEF	≥18	DAPA + SOC	SOC	direct cost	1 month	lifetime	3.5	QALY	1way,PSA
	Germany										3		
	Spain										3		
Nguyen et al. (2023)	United States	healthcare system	Markov	HFrEF	66.5	DAPA + SOC EMPA + SOC	SOC	direct cost	1 month	lifetime	3	QALY	1way,2way,PSA
Abushanab et al. (2024)	Qatar	healthcare	Markov	HFrEF and without T2D	66	DAPA + SOC	SOC	direct cost	3 months	lifetime	3	QALY	1way,PSA
Jiang et al. (2021)	China	healthcare system	Markov	HFrEF	66	DAPA + SOC EMPA + SOC	SOC	direct cost	3 months	10 years	5	QALY	1way,PSA
Yao et al. (2020)	China	healthcare payers	Markov	HFrEF	65	DAPA + SOC	SOC	direct cost	3 months	15 years	4.2	QALY	1way,2way,PSA
Liao et al. (2021)	South Korea	healthcare systems	Markov	HFrEF	66	DAPA + SOC	SOC	direct cost	1 month	15 years	3	QALY	1way,PSA
	Australia												
	China (Taiwan)												
	Japan												
	Singapore												
Bhatt et al. (2024)	United States	healthcare system	Markov	HF	一	DAPA + SOC	SOC	direct cost	1 month	lifetime	3	QALY	1way,2way,PSA
Rane et al. (2023)	United States	healthcare system	Markov	HFpEF	65	DAPA + SOC	EMPA + SOC	direct cost	1 year	lifetime	3	QALY	1way,PSA
Cheng et al. (2022)	China	healthcare system	Markov	HFrEF	65	DAPA + SOC	SOC	direct cost	1 month	lifetime	5	QALY	1way,PSA
Xu et al. (2023)	China	healthcare system	Markov	HFrEF	66	DAPA + SOC	SOC	direct cost	3 months	10 years	5	QALY	1way,PSA
Xia et al. (2022)	China	medical payers	Markov	HFpEF HFmrEF	72	DAPA + SOC EMPA + SOC	SOC	direct cost	3 months	10 years	一	QALY	1way,PSA
Lu et al. (2023b)	China	health system	Markov	HFrEF	≥18	DAPA + SOC SACU/VALS + SOC	EMPA + SOC	direct cost	3 months	30 years	5	QALY	1way,PSA

HFpEF, heart failure with preserved ejection fraction; HFmrEF, heart failure with mildly reduced ejection fraction; HFrEF, heart failure with reduced ejection fraction; HF, heart failure; T2D, Type 2 diabetes; DAPA, dapagliffozin; EMPA, empagliflozin; SOC, standard of care; CANA, canaglifozin; SACU/VALS, sacubitril/valsartan; QALY, quality-adjusted life-year; LY, life year; YoLS: years of life saved; 1way, one-way sensitivity analyses; 2way, two-way sensitivity analyses; PSA, probabilistic sensitivity analyses.

All studies used the Markov model in the decision-making process. Most studies compared dapagliflozin-SoC with SoC (standard of care) alone (n = 20) (Booth et al., 2023; Kim et al., 2024; Abdelhamid et al., 2022; Gil-Rojas et al., 2022; Krittayaphong and Permsuwan, 2021; Mendoza et al., 2021; Tang and Sang, 2023; Miller et al., 2023; Lin et al., 2023; Savira et al., 2021; Parizo et al., 2021; Isaza et al., 2021; Davis et al., 2024; McEwan et al., 2020; Abushanab et al., 2024; Yao et al., 2020; Liao et al., 2021; Bhatt et al., 2024; Chen et al., 2022; Xu et al., 2023). Four studies compared dapagliflozin-SoC and empagliflozin-SoC against SoC alone (Lu H. et al., 2023; Nguyen et al., 2023; Jiang et al., 2021; Xia et al., 2022). Two studies compared dapagliflozin-SoC against empagliflozin-SoC (Nechi et al., 2023; Rane et al., 2023). One study compared dapagliflozin-Soc, canagliflozin-SoC, empagliflozin-SoC against SoC alone (Kongmalai et al., 2025). One study compared dapagliflozin- Soc, sacubitril/valsartan-SoC against empagliflozin-SoC (Lu D. et al., 2023). The time horizon of most of the studies was lifetime (n = 19) (Booth et al., 2023; Kim et al., 2024; Abdelhamid et al., 2022; Kongmalai et al., 2025; Krittayaphong and Permsuwan, 2021; Mendoza et al., 2021; Miller et al., 2023; Nechi et al., 2023; Lin et al., 2023; Savira et al., 2021; Parizo et al., 2021; Isaza et al., 2021; Davis et al., 2024; McEwan et al., 2020; Nguyen et al., 2023; Abushanab et al., 2024; Bhatt et al., 2024; Rane et al., 2023; Chen et al., 2022), three studies were 15 years (Tang and Sang, 2023; Yao et al., 2020; Liao et al., 2021), three studies were 10 years (Jiang et al., 2021; Xu et al., 2023; Xia et al., 2022), one study was 5 years (Gil-Rojas et al., 2022), one study was 20 years (Lu H. et al., 2023), and one study was 30 years (Lu D. et al., 2023). The length of the cycles was 1 month (n = 14) (Booth et al., 2023; Kim et al., 2024; Abdelhamid et al., 2022; Gil-Rojas et al., 2022; Miller et al., 2023; Lin et al., 2023; Parizo et al., 2021; Isaza et al., 2021; Davis et al., 2024; McEwan et al., 2020; Nguyen et al., 2023; Liao et al., 2021; Bhatt et al., 2024; Chen et al., 2022), 3 months (n = 10) (Lu H. et al., 2023; Kongmalai et al., 2025; Krittayaphong and Permsuwan, 2021; Tang and Sang, 2023; Abushanab et al., 2024; Jiang et al., 2021; Yao et al., 2020; Xu et al., 2023; Xia et al., 2022; Lu D. et al., 2023) and 1 year (n = 4) (Mendoza et al., 2021; Nechi et al., 2023; Savira et al., 2021; Rane et al., 2023).

### Reporting quality assessment

The CHEERS checklist was used to evaluate the quality of the study, and the results are shown in Table 2. All studies scored above 75 points, so they belong to the category of high-quality research. The following three items from the CHEERS checklist were missing from all studies: health economic analysis plan, approach to engagement with patients and others affected by the study, and effect of engagement with patients and others affected by the study respectively. Two studies did not mention discount rate (Abdelhamid et al., 2022; Xia et al., 2022). Nine studies did not mention characterizing heterogeneity (Lu H. et al., 2023; Kongmalai et al., 2025; Nechi et al., 2023; Lin et al., 2023; Bhatt et al., 2024; Chen et al., 2022; Xu et al., 2023; Xia et al., 2022; Lu D. et al., 2023). Four studies did not mention characterizing uncertainty and effect of uncertainty (Kim et al., 2024; Bhatt et al., 2024; Chen et al., 2022; Xia et al., 2022). One study did not mention study parameters (Lu H. et al., 2023). Eight studies did not mention source of funding (Lu H. et al., 2023; Krittayaphong and Permsuwan, 2021; Lin et al., 2023; Jiang et al., 2021; Rane et al., 2023; Chen et al., 2022; Xu et al., 2023; Xia et al., 2022).

**TABLE 2:** Reporting quality of the economic evaluations (as assessed by the CHEERS statement).

Item no.	Section/item	Booth et al. (2023)	Lu et al. (2023a)	Kim et al. (2024)	Abdelhamid et al. (2022)	Gil-Rojas et al. (2022)	Kongmalai et al. (2025)	Krittayaphong and Permsuwan, (2021)	Mendoza et al. (2021)	Tang and Sang, (2023)	Miller et al. (2023)	Nechi et al. (2023)
1	Title	1	1	1	1	1	1	1	1	1	1	1
2	Abstract	1	1	1	1	1	1	1	1	1	1	1
3	Background and objectives	1	1	1	1	1	1	1	1	1	1	1
4	Health economic analysis plan	0	0	0	0	0	0	0	0	0	0	0
5	Study population	1	1	1	1	1	1	1	1	1	1	1
6	Setting and location	1	1	1	1	1	1	1	1	1	1	1
7	Comparators	1	1	1	1	1	1	1	1	1	1	1
8	Perspective	1	1	1	1	1	1	1	1	1	1	1
9	Time horizon	1	1	1	1	1	1	1	1	1	1	1
10	Discount rate	1	1	1	0	1	1	1	1	1	1	1
11	Selection of outcomes	1	1	1	1	1	1	1	1	1	1	1
12	Measurement of outcomes	1	1	1	1	1	1	1	1	1	1	1
13	Valuation of outcomes	1	1	1	1	1	1	1	1	1	1	1
14	Measurement and valuation of resources and costs	1	1	1	1	1	1	1	1	1	1	1
15	Currency, price date, and conversion	1	1	1	1	1	1	1	1	1	1	1
16	Rationale and description of mode	1	1	1	1	1	1	1	1	1	1	1
17	Analytics and assumptions	1	1	1	1	1	1	1	1	1	1	1
18	Characterizing heterogeneity	1	0	1	1	1	0	1	1	1	1	0
19	Characterizing distributional effects	1	1	1	1	1	1	1	1	1	1	1
20	Characterizing uncertainty	1	1	0	1	1	1	1	1	1	1	1
21	Approach to engagement with patients and others affected by the study	0	0	0	0	0	0	0	0	0	0	0
22	Study parameters	1	0	1	1	1	1	1	1	1	1	1
23	Summary of main results	1	1	1	1	1	1	1	1	1	1	1
24	Effect of uncertainty	1	1	0	1	1	1	1	1	1	1	1
25	Effect of engagement with patients and others affected by the study	0	0	0	0	0	0	0	0	0	0	0
26	Study findings, limitations, generalisability, and currentknowledge	1	1	1	1	1	1	1	1	1	1	1
27	Source of funding	1	0	1	1	1	1	0	1	1	1	1
28	onflicts of interest	1	1	1	1	1	1	1	1	1	1	1
Overall quality		1	1	1	1	1	1	1	1	1	1	1

“1” meets the quality assessment criteria; “0” does not fully conform to the quality assessment criteria; CHEERS, consolidated health economic evaluation reporting standards.

### Cost-effectiveness analysis

The overview of the economic evaluation outcomes is summarized in Table 3.

**TABLE 3:** Overview of economic evaluation outcomes of included studies.

Author	Country	Target population	Discount year	Intervention	Comparison	Costs (original currency; mean)		QALY		△Cost	△QALY	ICER (2024 US$ per QALY)
						I	C	I	C			
Booth et al. (2023)	United Kingdom	HFpEF HFmrEF	2021	DAPA + SOC	SOC	£12 062	£10 267	4.865	4.633	£1 795	0.231	£7 761 ($12909.35)
	Germany	HFpEF HFmrEF	2021			€14 496	€11 938	5.823	5.554	€2 558	0.268	€9 540 ($14146.40)
	Spain	HFpEF HFmrEF	2021			€12 116	€10 725	5.448	5.188	€1 391	0.260	€5 343 ($9532.11)
Lu et al. (2023a)	China	HFpEF	2022	DAPA + SOC	SOC	$8 153.14	$6 151	7.43	7.09	$2 002.13	0.34	$5 907.79 ($6263.77)
				EMPA + SOC		$8 250.55	$6 626.97	7.32	6.88	$1 623.58	0.44	$3 691.56 ($3914.00)
Kim et al. (2024)	South Korea	HF(LVE≤ 40%)	2022	DAPA + SOC	SOC	$20 142	$18 276	6.63	6.06	$1 866	0.57	$3 279 ($3476.58)
		HF(LVEF >40%)				$21 134	$18 481	8.34	8.03	$2 653	0.32	$8 383 ($8888.13)
		HF (Overall)				$20 514	$18 353	7.27	6.80	$2 161	0.47	$4 557 ($4831.59)
Abdelhamid et al. (2022)	Egypt	HFrEF	2021	DAPA + SOC	SOC	EGP 47 901	EGP 34 377	4.57	4.20	EGP 13 524	0.371	EGP36 449 ($13031.83)
Gil-Rojas et al. (2022)	Colombia	HFrEF	2020	DAPA + SOC	SOC	$4 611.2	$3 808.3	2.689	2.544	$802.9	0.135	$5 945.9 ($7048.82)
Kongmalai et al. (2025)	Thailand	T2D-HF	2022	DAPA + SOC	SOC	$9 628	$6 082	4.27	3.73	$3 547	0.54	$6 430 ($6817.45)
				EMPA + SOC		$8 776		3.79		$2 694	0.06	$48 952 ($51901.65)
				CANA + SOC		$11 682		4.95		$5 600	1.22	$4 632 ($4911.11)
				SGLT-2+SOC		$9 559		4.14		$3 477	0.41	$8 480 ($8990.97)
Krittayaphong and Permsuwan, (2021)	Thailand	HFrEF	2019	DAPA + SOC	SOC	THB54 405	THB17 442	6.92	6.33	THB36 963	0.60	THB62 090 ($5449.37)
Mendoza et al. (2021)	Philippines	HFrEF	2019	DAPA + SOC	SOC	——	——	——	——	——	——	PHP177 868 ($10706.32)
Tang and Sang, (2023)	China	HFpEF, HFmrEF	2022	DAPA + SOC	SOC	$7 245.77	$5 407.55	6.00	5.84	$1 838.22	0.15	$11 865.33 ($12580.29)
Miller et al. (2023)	United Kingdom	HFrEF	2019	DAPA + SOC	SOC	£16 660 (after12months)	£13 224	4.614	4.023	£3 436	0.591	£5 820.51 ($10257.77)
						£16 912 (immediate)	£13 224	4.662	4.023	£3 688	0.639	£5 778.68 ($10184.05)
	Canada					$53 839 (after12months)	$47 096	5.079	4.373	$6 743	0.706	$9 553 ($11472.82)
						$53 940 (immediate)	$47 096	5.138	4.373	$6 844	0.765	$8 945 ($10742.63)
	Germany					€26 222 (after12months)	€23 020	4.721	4.105	€3 202	0.616	$5 193 ($8081.32)
						€26 257 (immediate)	€23 020	4.772	4.105	€3 237	0.667	$4 853 ($7552.22)
	Spain					€25 469 (after12months)	€19 832	4.721	4.105	€5 637	0.616	€9 144 ($16937.29)
						€25 809 (immediate)	€19 832	4.772	4.105	€5 977	0.667	€8 963 ($16602.02)
Nechi et al. (2023)	United States	HFrEF	2022	DAPA + SOC	EMPA + SOC	$221 785	$184 101	4.776	3.935	$37 684	0.841	$44 763 ($47460.24)
Lu et al. (2023a)	China	HFpEF HFmrEF	2022	DAPA + SOC	SOC	$9 807.5	$8 256.5	6.46	6.32	$1 551	0.15	$10 615.87 ($11255.54)
Savira et al. (2021)	Australia	HFrEF	2020	DAPA + SOC	SOC	A$28 445.855	A$24 753.415	2.789	2.502	A$3 692.440	0.288	A$12 842 ($15224.09)
Parizo et al. (2021)	United States	HFrEF	2020	DAPA + SOC	SOC	$183 583	$145 371	5.7	5.2	$38 212	0.2	$83 650 ($99166.39)
Isaza et al. (2021)	United States	HFrEF	2020	DAPA + SOC	SOC	$193 400	$150 600	5.36	4.73	$42 800	0.63	$68 300 ($80969.09)
Davis et al. (2024)	United Kingdom	HF	2022	DAPA + SOC	SOC	£14 753	£12 805	5.184	4.882	£1 948	0.301	£6 470 ($10209.61)
McEwan et al. (2020)	United Kingdom	HFrEF	2019	DAPA + SOC	SOC	£16 408	£13 628	4.61	4.13	£2 780	0.48	£5 822 ($10260.39)
	Germany					€25 328	€22 647	4.72	4.22	€2 681	0.50	€5 379 ($8370.77)
	Spain					€24 330	€19 642	4.72	4.22	€4 688	0.50	€9 406 ($17422.58)
Nguyen et al. (2023)	United States	HFrEF	2022	DAPA + SOC	SOC	$108 378	$87 095	4.88	4.50	$21 284	0.37	$56 782 ($60203.45)
				EMPA + SOC	SOC	$102 854		4.68		$15 760	0.18	$89 258 ($94636.32)
				DAPA + SOC	EMPA + SOC	$108 378	$102 854	4.88	4.68	$5 524	0.20	$27 861 ($29539.79)
Abushanab et al. (2024)	Qatar	HFrEF and without T2D	2023	DAPA + SOC	SOC	QAR43 184	QAR 42 413	8.496	7.865	QAR 771	0.6	QAR 5 212 ($2796.96)
Jiang et al. (2021)	China	HFrEF	2020	DAPA + SOC	SOC	$4 870.68	$3 596.25	3.87	3.64	$1 274.43	0.23	$5 541 ($6568.81)
				EMPA + SOC		$5 021.93	$4 118.86	3.66	3.53	$903.07	0.13	$6 946.69 ($8235.24)
Yao et al. (2020)	China	HFrEF	2017	DAPA + SOC	SOC	$5 858.4	$4 436.6	4.82	4.44	$1 421.8	0.38	$3 732.3 ($4672.43)
Liao et al. (2021)	South Korea	HFrEF	2020	DAPA + SOC	SOC	$17 577	$13 277	9.56	8.74	$4 300	0.81	$5 277 ($6255.84)
	Australia					$59 126	$50 745	9.85	9.01	$8 381	0.84	$9 980 ($11831.21)
	China (Taiwan)					$87 805	$76 501	11.03	10.09	$11 304	0.94	$12 305 ($14587.48)
	Japan					$49 064	$35 453	9.56	8.74	$13 611	0.81	$16 705 ($19803.64)
	Singapore					$160 525	$140 153	10.29	9.42	$20 372	0.88	$23 227 ($27535.42)
Bhatt et al. (2024)	United States	HF	2020	DAPA + SOC	SOC	$154 512	$109 003	6.57	6.04	$45 509	0.53	$85 554 ($101423.57)
Rane et al. (2023)	United States	HFpEF	2022	DAPA + SOC	EMPA + SOC	$191 202	$161 306	4.953	4.143	$29 896	0.810	$36 902 ($39125.56)
Chen et al. (2022)	China	HFrEF	2021	DAPA + SOC	SOC	¥77 893.14	¥72 175.57	11.9	11.2	¥5 717.57	0.7	¥8 821.32 ($2569.76)
Xu et al. (2023)	China	HFrEF	2022	DAPA + SOC	SOC	¥33 662.91	¥23 686.9	4.80	4.34	¥9 976.01	0.46	¥21 649.33 ($6173.24)
Xia et al. (2022)	China	HFpEF, HFmrEF	2019	DAPA + SOC	SOC	¥57 546	¥49 411	4.13	4.05	¥8 135	0.082	¥98 722 ($29983.58)
				EMPA + SOC		¥56 345	¥48 648	4.13	4.05	¥7 697	0.078	¥98 248 ($29839.62)
Lu et al. (2023b)	China	HFrEF	2022	DAPA + SOC	EMPA + SOC	¥32 163.62	¥27 303.95	1.35	1.11	¥4 859.67	0.24	¥20 248.63 ($5773.83)
				SACU/VALS + SOC		¥37 564.17	¥27 303.95	1.54	1.11	¥10 260.22	0.43	¥23 860.98 ($6803.88)

HFpEF, heart failure with preserved ejection fraction; HFmrEF, heart failure with mildly reduced ejection fraction; HFrEF, heart failure with reduced ejection fraction; HF, heart failure; T2D, Type 2 diabetes; DAPA, dapagliffozin; EMPA, empagliflozin; SOC, standard of care; CANA, canaglifozin; SACU/VALS, sacubitril/valsartan; ICER, incremental cost effectiveness ratios; PSA, probabilistic sensitivity analyses; WTP, willingness-To-pay.

Eighteen studies provided economic evaluation for HFrEF. The studies were conducted in 15 countries (China, UK, United States, Japan, Korea, Singapore, Thailand, Australia, Egypt, Colombia, Philippines, Qatari, Canadian, German, and Spanish). All the studies indicated that add-on dapagliflozin was cost-effective for HFrEF compared to standard of care alone in these countries. One study (Miller et al., 2023) compared the cost-effectiveness of immediate and delayed 12-month using dapagliflozin in patients with HFrEF from a healthcare perspective in the UK, Canada, Germany, and Spain. The results showed that both immediate use and 12-month delayed use of dapagliflozin were cost-effective, but starting treatment immediately was more cost-effective. In addition, three studies (Nechi et al., 2023; Nguyen et al., 2023; Jiang et al., 2021) (two in the US and one in China) compared cost-effectiveness of dapagliflozin with empagliflozin for treatment of HFrEF. The results all showed that dapagliflozin was more cost-effective than empagliflozin for treatment of HFrEF.

Two studies provided economic evaluation for HFpEF. Both results suggest that dapagliflozin was cost-effective for HFpEF. One study (Lu H. et al., 2023) evaluated the cost-effectiveness of SGLT2 inhibitors (dapagliflozin or empagliflozin) for treatment of HFpEF from the perspective of the Chinese health system. The results showed that dapagliflozin-SoC or empagliflozin-SoC were more cost-effective than SoC alone for treatment of HFpEF. Another study (Rane et al., 2023) compared the cost-effectiveness of dapagliflozin vs. empagliflozin for treatment of HFpEF from the US healthcare system perspective. The results suggested that dapagliflozin-SoC is more cost-effective than empagliflozin-SoC, and its uptake may improve long-term outcomes of HFpEF.

Four studies provided economic evaluation for HFpEF/HFmrEF (LVEF >40%). One study (Booth et al., 2023) was conducted in 3 countries (UK, German, and Spanish). Three studies (Tang and Sang, 2023; Lin et al., 2023; Xia et al., 2022) were conducted in China. All the studies indicated that add-on dapagliflozin was cost effective for HFpEF/HFmrEF compared to standard of care alone in these countries.

Four studies provided economic evaluation for HF (across the entire EF spectrum). Three studies (Kim et al., 2024; Davis et al., 2024; Bhatt et al., 2024) (one in Korea, one in UK, one in US) indicated that add-on dapagliflozin was cost effective for HF across the entire EF spectrum compared to standard of care alone. Among them, the study (Kim et al., 2024) in Korea showed that the cost-effectiveness of patients with left ventricular EF (LVEF)≤40% (ICER: 3476.58 USD/QALY) was more pronounced than LVEF >40% (ICER: 8888.13 USD/QALY). But one study (Kongmalai et al., 2025) in Thailand suggested that it is not cost-effective to add SGLT2i (dapagliflozin, empagliflozin, canagliflozin) to the standard treatment for HF with type 2 diabetes (T2D) patients. The ICERs for dapagliflozin, empagliflozin, canagliflozin and overall SGLT2i were US$6817.45, US$ 51901.65, US$4911.11and US$8990.97 per QALY gained, respectively. Thailand’s willingness-to-pay threshold of US$4564 per QALY gained. To be cost-effective, the costs of dapagliflozin, empagliflozin, canagliflozin and overall SGLT-2i should be reduced by 38.2%, 90.2%,2.3% and 55.6%, respectively.

### Subgroup analysis

Nine studies carried out subgroup analysis of diabetes (Gil-Rojas et al., 2022; Krittayaphong and Permsuwan, 2021; Mendoza et al., 2021; Savira et al., 2021; Parizo et al., 2021; Isaza et al., 2021; McEwan et al., 2020; Nguyen et al., 2023; Jiang et al., 2021) and all the research results showed that dapagliflozin was cost-effective for HErEF diabetes patients and non-diabetes patients. Among them, seven studies (Gil-Rojas et al., 2022; Krittayaphong and Permsuwan, 2021; Mendoza et al., 2021; Parizo et al., 2021; Isaza et al., 2021; Nguyen et al., 2023; Jiang et al., 2021) showed that dapagliflozin was more cost-effective for patients with diabetes than for patients without diabetes. The results of the other two studies (Savira et al., 2021; McEwan et al., 2020) are different. One study (Savira et al., 2021) showed that dapagliflozin was more cost-effective for HErEF patients with non-diabetes than for patients with diabetes within a lifetime horizon, but when the time horizon was limited to 2 years, the trend of cost-effectiveness was reversed, and dapagliflozin was more cost-effective for HErEF patients with diabetes than for patients with non-diabetes. The details are shown in Table 4.

**TABLE 4:** Subgroup analyses of diabetes status.

Author	Country	ICER	
		With diabetes	Without diabetes
Gil-Rojas et al. (2022)	Colombia	$4 881	$6 867
Krittayaphong and Permsuwan, (2021)	Thailand	THB47 613	THB68 304
Mendoza et al. (2021)	Philippines	PHP132 582	PHP278 286
Savira et al. (2021)	Australia	Llife time:A$13 234	Life time:$A12 386
		Two years:A $32 098	Two years:A $42 178
Parizo et al. (2021)	United States	$79 726	$85 420
Isaza et al. (2021)	United States	$66 800	$69 600
McEwan et al. (2020)	United Kingdom	£6 350	£5 419
	Spain	€ 9 991	€ 8 964
	Germany	€ 6 008	€ 4 893
Nguyen et al. (2023)	United States	$50 740	$65 473
Jiang et al. (2021)	China	$4 411	$6 790

ICER, incremental cost effectiveness ratios.

In addition, one study (Nguyen et al., 2023) conducted a subgroup analysis of chronic kidney disease, and the results showed that dapagliflozin was cost-effective for both HErEF patients with and without chronic kidney disease, with non-chronic kidney disease patients being more cost-effective. One study (Parizo et al., 2021) conducted a subgroup analysis of health impairment caused by heart failure, and the results showed that dapagliflozin was cost-effective for both mild and moderate health impairment patients with HErEF, with mild health impairment patients being more cost-effective.

### Uncertainty analysis

One-way sensitivity and probabilistic sensitivity analyses were performed in all studies, and two-way sensitivity analyses were also used in five studies (Parizo et al., 2021; Davis et al., 2024; Nguyen et al., 2023; Yao et al., 2020; Bhatt et al., 2024). Seventeen studies (Booth et al., 2023; Lu H. et al., 2023; Kim et al., 2024; Abdelhamid et al., 2022; Gil-Rojas et al., 2022; Krittayaphong and Permsuwan, 2021; Mendoza et al., 2021; Miller et al., 2023; Nechi et al., 2023; Lin et al., 2023; Isaza et al., 2021; Nguyen et al., 2023; Abushanab et al., 2024; Yao et al., 2020; Liao et al., 2021; Bhatt et al., 2024; Rane et al., 2023) reported that the main factor affecting ICER is the cost of dapagliflozin. Eight studies (Booth et al., 2023; Kim et al., 2024; Abdelhamid et al., 2022; Gil-Rojas et al., 2022; Krittayaphong and Permsuwan, 2021; Mendoza et al., 2021; Miller et al., 2023; Rane et al., 2023) showed that when the cost increases to the upper limit, ICER is still below the WTP threshold. One study showed (Yao et al., 2020) that when the cost increased to the upper limit, ICER was higher than China’s *per capita* GDP in 2017 ($8573.4), but it is still less than three times the *per capita* GDP.

Twelve studies (Lu H. et al., 2023; Kongmalai et al., 2025; Tang and Sang, 2023; Lin et al., 2023; Parizo et al., 2021; Nguyen et al., 2023; Abushanab et al., 2024; Jiang et al., 2021; Yao et al., 2020; Liao et al., 2021; Xu et al., 2023; Xia et al., 2022) indicated that cardiovascular mortality is the most important influencing factor of ICER. In one study (Lu H. et al., 2023), dapagliflozin showed satisfactory cost-effectiveness when the corresponding ICER remained below the WTP threshold when the upper limit of cardiovascular mortality was reached. However, another study (Kongmalai et al., 2025) showed that when the upper limit of cardiovascular mortality was reached, dapagliflozin resulted in an increase in QALYs, with an ICERs of US$ 6817.45 per additional QALY, which is above the WTP threshold and is not cost-effective.

Other studies suggested that key drivers of cost-effectiveness also include the duration of dapagliflozin effectiveness (Parizo et al., 2021; Isaza et al., 2021), the interaction of LVEF changes with changes in other characteristics (Davis et al., 2024), the discount rate and the HR (risk ratio) of hospital admission for HF (Chen et al., 2022).

## Discussion

This systematic review provides the most extensive synthesis to date on the cost-effectiveness of dapagliflozin for heart failure, encompassing all LVEF subtypes (HFrEF, HFmrEF, and HFpEF). By integrating 28 studies from 15 countries, we address a critical evidence gap left by prior reviews limited to HFrEF populations. Previous studies (Wu et al., 2022; Mohammadnezhad et al., 2022; Rezapour et al., 2023) focused on patients with HFrEF, and this study included HFpEF and HFmrEF. This study also compares the cost-effectiveness of dapagliflozin with empagliflozin. In addition, previous studies (Wu et al., 2022; Mohammadnezhad et al., 2022; Rezapour et al., 2023) were included up to 2021 or 2022, and this study adds 15 articles published in 2023 and 2024.

This study identified 28 economic evaluations of dapagliflozin for the treatment of HF from 15 countries, and the results showed that dapagliflozin for the treatment of HF was cost-effective in most countries. Only one study in Thailand (Kongmalai et al., 2025) showed that dapagliflozin was not cost-effective in patients with T2D-HF. Another study (Krittayaphong and Permsuwan, 2021), also in Thailand, showed that dapagliflozin was cost-effective for HFrEF. In addition, in three studies in the UK (Booth et al., 2023; Miller et al., 2023; McEwan et al., 2020), ICER ($10184.05, $10260.39 per QALY) in two studies in patients with HFrEF were lower than ICER ($12909.35 per QALY) in one study in patients with HFpEF/HFmrEF. In the eight studies in China (Tang and Sang, 2023; Lin et al., 2023; Jiang et al., 2021; Yao et al., 2020; Chen et al., 2022; Xu et al., 2023; Xia et al., 2022; Lu D. et al., 2023), ICER ($6568.81, $4672.43, $2569.76, $6173.24, $5773.83 per QALY) in five HFrEF studies were also significantly lower than ICER ($12580.29, $11255.54, $29983.58 per QALY) in the three HFpEF/HFmrEF studies. Because these studies were conducted separately, we cannot directly conclude that dapagliflozin in patients with HFrEF is more cost-effective than HFpEF/HFmrEF. But a South Korean study (Kim et al., 2024) confirms this with LVEF≤40% (ICER: $3476.58 per QALY) and LVEF >40% (ICER:$8888.13 per QALY). So, we can conclude that the cost-effectiveness of dapagliflozin in patients with LVEF≤40% (HErEF) was more pronounced than LVEF >40% (HFpEF and HFmrEF).

Substantial variability in ICER across countries aligns with prior studies, primarily driven by differences in drug pricing policies, healthcare infrastructure, and national willingness-to-pay thresholds (Mcdonagh et al., 2024; Sarafidis et al., 2021; Packer et al., 2020). In the study of patients with HFrEF, the highest ICER was in the United States (Bhatt et al., 2024), with the ICER of $101423.57 per QALY obtained. The lowest ICER was in China (Chen et al., 2022), with the ICER of $2569.76 per QALY earned. The main reason for the large variance in ICER is the large variance in cost of dapagliflozin in different countries, which due to the differences in healthcare policies, economic and medical levels among different countries. The ICER for Taiwan (Davis et al., 2024), also China, was $14587.48 per QALY. This proves the variance between different regions of the same country. Due to the differences of public health policies and economic level between the two regions in the same country, we should consider the heterogeneity in different regions when we evaluate the cost effectiveness.

One study (Miller et al., 2023) conducted in the UK, Canada, Germany, and Spain compared the cost-effectiveness of immediate and delayed 12-month treatment with dapagliflozin in patients with HFrEF. Results from four countries consistently suggest that both immediate and 12-month delay in initiation of dapagliflozin is cost-effective. However, it is more cost-effective to start dapagliflozin immediately than to delay it for 12 months. The results provide a useful reference for the use of dapagliflozin in clinical practice.

Four studies (Nechi et al., 2023; Nguyen et al., 2023; Rane et al., 2023; Lu D. et al., 2023) (two HFrEF and one HFpEF in the US and one HFrEF in China) conducted cost-effective studies comparing dapagliflozin with empagliflozin, and all showed that dapagliflozin was more cost-effective than empagliflozin. However, the results are not absolute, and the results may vary depending on the influencing factors. As a sensitivity analysis for one of the studies (Nguyen et al., 2023), dapagliflozin-SoC is not cost-effective compared to empagliflozin-SoC if the HR (hazard ratio) for cardiovascular death with dapagliflozin >0.965 or empagliflozin for cardiovascular death <0.768. In addition, the uncertainty analysis showed that the main influencing factor of ICER was the cost of dapagliflozin. Dapagliflozin’s patent for treating heart failure indications expires in 2025, and generic agents are likely to drive price competition, significantly reducing drug costs, making dapagliflozin more cost-effective for heart failure. Similarly, once the patent for empagliflozin expires, the price is expected to decrease as well.

In the diabetes subgroup analyses, results differ on the question of which is more cost-effective for HErEF with diabetes and without diabetes. The results of one of the studies (Savira et al., 2021) showed that non-diabetic patients were more cost-effective over a lifetime time horizon. Because people without diabetes live longer, dapagliflozin prevents more acute hospitalizations. When the time horizon was limited to 2 years, the trend towards cost-effectiveness reversed, and it was more cost-effective for diabetes patients because dapagliflozin provided more benefits to diabetes patients and prevented more acute events. In fact, HErEF patients with diabetes face a higher risk of CV and renal events and have higher healthcare management costs compared to those without diabetes. Therefore, it is difficult to draw conclusions about which is more cost-effective.

There are some limitations to our review. First, although this is the most comprehensive systematic review of the cost-effectiveness of dapagliflozin in HF, the included studies focused on patients with HErEF and fewer studies on patients with HFpEF and HFmrEF. Second, no studies comparing dapagliflozin with other SGLT2 inhibitors (canagliflozin and Sotagliflozin) were identified, and it was not possible to compare the cost-effectiveness of dapagliflozin with other SGLT2 inhibitors. Third, due to inherent limitations of applying quantitative statistical tests to model-based economic evaluations, meta-analysis of study data was not possible, and studies were limited to systematic reviews.

## Conclusion

We conducted a comprehensive systematic review of the economic evaluation of dapagliflozin for the treatment of heart failure with all ejection fractions. Based on the results of the included studies, dapagliflozin is cost-effective in all studies except for one study in Thailand. The cost-effectiveness of dapagliflozin in patients with LVEF≤40% (HErEF) was more pronounced than LVEF >40% (HFpEF and HFmrEF). Compared to empagliflozin, dapagliflozin may be more cost-effective. The cost of dapagliflozin and cardiovascular mortality were the main influencing factors for ICER, followed by the HR of hospital admission for HF, the discount rate and the interaction of LVEF changes with changes in other characteristics.

## Data Availability

The original contributions presented in the study are included in the article/Supplementary Material, further inquiries can be directed to the corresponding author.
