# Supplementary material for: Cost-effectiveness of dapagliflozin for the treatment of heart failure: a systematic review
Source: Front Pharmacol. 2025 May 23;16:1572289. doi: 10.3389/fphar.2025.1572289 (PMC12141269; doi:10.3389/fphar.2025.1572289)
Supplement: Supplementary file 1 [file Table1.docx]

**PubMed**

| **Search strategy** | | **Item found** |
| --- | --- | --- |
| 1 | "dapagliflozin" OR " forxiga" OR " farxiga" OR " BMS-512148" OR OR " BMS512148" | 3356 |
| #2 | "cost-effectiveness" OR "cost-utility" OR "cost-benefit" OR "cost-minimization" OR "pharmacoeconomic study" OR "economic study" OR "pharmacoeconomic evaluation" OR "economic evaluation" | 153126 |
| #3 | "heart failure" OR "cardiac failure" OR "myocardial failure" OR "cardiac dysfunction" OR "heart dysfunction" OR "myocardial dysfunction" | 300376 |
| #4 | #1,#2,#3 | 53 |

**web of science**

| **Search strategy** | | **Item found** |
| --- | --- | --- |
| #1 | "dapagliflozin" OR " forxiga" OR " farxiga" OR " BMS-512148" OR OR " BMS512148" | 5611 |
| #2 | "cost-effectiveness" OR "cost-utility" OR "cost-benefit" OR "cost-minimization" OR "pharmacoeconomic study" OR "economic study" OR "pharmacoeconomic evaluation" OR "economic evaluation" | 198482 |
| #3 | "heart failure" OR "cardiac failure" OR "myocardial failure" OR "cardiac dysfunction" OR "heart dysfunction" OR "myocardial dysfunction" | 351068 |
| #4 | #1,#2,#3 | 77 |

**Embase**

| **Search strategy** | | **Item found** |
| --- | --- | --- |
| #1 | "dapagliflozin" OR " forxiga" OR " farxiga" OR " BMS-512148" OR OR " BMS512148" | 5766 |
| #2 | "cost-effectiveness" OR "cost-utility" OR "cost-benefit" OR "cost-minimization" OR "pharmacoeconomic study" OR "economic study" OR "pharmacoeconomic evaluation" OR "economic evaluation" | 149604 |
| #3 | "heart failure" OR "cardiac failure" OR "myocardial failure" OR "cardiac dysfunction" OR "heart dysfunction" OR "myocardial dysfunction" | 428160 |
| #4 | #1,#2,#3 | 79 |

**Cochrane**

| **Search strategy** | | **Item found** |
| --- | --- | --- |
| #1 | "dapagliflozin" OR " forxiga" OR " farxiga" OR " BMS-512148" OR OR " BMS512148" | 2321 |
| #2 | "cost-effectiveness" OR "cost-utility" OR "cost-benefit" OR "cost-minimization" OR "pharmacoeconomic study" OR "economic study" OR "pharmacoeconomic evaluation" OR "economic evaluation" | 43900 |
| #3 | "heart failure" OR "cardiac failure" OR "myocardial failure" OR "cardiac dysfunction" OR "heart dysfunction" OR "myocardial dysfunction" | 39692 |
| #4 | #1,#2,#3 | 27 |

**Chinese database:**

**China National Knowledge Infrastructure (CNKI)**

| Search strategy | Item found |
| --- | --- |
| (TKA=心力衰竭OR TKA=心肌衰竭 OR TKA=心功能障碍 OR TKA=心肌功能障碍) AND (TKA=费用 OR TKA=成本 OR TKA=经济 OR TKA=负担 OR TKA=卫生支出 OR TKA=卫生费用) AND (TKA=达格列净) | 9 |

**Chinese database: Wanfang Data**

| Search strategy | Item found |
| --- | --- |
| [全部字段=(心力衰竭OR心肌衰竭 0R心功能障碍 OR心肌功能障碍) AND 全部字段=(费用OR 成本OR 经济 OR 负担 OR 卫生支出 OR 卫生费用) AND 全部字段=(达格列净)](http://med.wanfangdata.com.cn/Paper/Search?q=((((%E4%B8%99%E5%9E%8B%E8%82%9D%E7%82%8E)%20OR%20%E4%B8%99%E8%82%9D))%20AND%20((((((%E8%B4%B9%E7%94%A8)%20OR%20%E6%88%90%E6%9C%AC)%20OR%20%E7%BB%8F%E6%B5%8E)%20OR%20%E8%B4%9F%E6%8B%85)%20OR%20%E5%8D%AB%E7%94%9F%E6%94%AF%E5%87%BA)))%20AND%20(((%E8%89%BE%E5%B0%94%E5%B7%B4%E9%9F%A6)%20AND%20%E6%A0%BC%E6%8B%89%E7%91%9E%E9%9F%A6))) | 14 |

**Chinese database: the Chongqin VIP**

| Search strategy | Item found |
| --- | --- |
| M=(心力衰竭 OR 心肌衰竭 OR 心功能障碍 OR 心肌功能障碍) AND (费用 OR 成本 OR 经济 OR 负担 OR 卫生支出 OR 卫生费用) AND (达格列净) | 6 |
